# The genome sequence of the nematode *Caenorhabditis drosophilae *(Rhabditida, Rhabditidae) (Kiontke, 1997)

**DOI:** 10.12688/wellcomeopenres.22416.1

**Published:** 2024-05-24

**Authors:** Manuela Kieninger, Lewis Stevens, Joanna C. Collins, Wellcome Sanger Institute Tree of Life Management, Samples and Laboratory team, Wellcome Sanger Institute Tree of Life Core Informatics team, Wellcome Sanger Institute Scientific Operations: Sequencing Operations, Mark Blaxter

**Affiliations:** 1 Tree of Life, Wellcome Sanger Institute, Hinxton, England, UK

**Keywords:** Caenorhabditis drosophilae, genome sequence, chromosomal, Rhabditida

## Abstract

We present a genome assembly of the free-living nematode *Caenorhabditis drosophilae* (Nematoda; Chromadorea; Rhabditida; Rhabditidae). The genome sequence is 51.3 megabases in span. Most of the assembly is scaffolded into six chromosomal pseudomolecules, including the X sex chromosome. The mitochondrial genome has also been assembled and is 15.15 kilobases in length.

## Species taxonomy

Eukaryota; Opisthokonta; Metazoa; Eumetazoa; Bilateria; Protostomia; Ecdysozoa; Nematoda; Chromadorea; Rhabditida; Rhabditina; Rhabditomorpha; Rhabditoidea; Rhabditidae; Peloderinae; *Caenorhabditis*; *Caenorhabditis drosophilae* ( Kiontke, 1997).

## Background

*Caenorhabditis drosophilae* is a free-living, gonochoristic nematode species that was originally isolated from its phoretic host *Drosophila nigrospiculata*, a fly species that feeds on rot of saguaro cactus, *Carnegiea gigantea*, in Arizona, USA ( Kiontke, 1997; Kiontke, 1999). *C. drosophilae* are bacterivorous and can be maintained on agar plates seeded with *Escherichia coli*, but presumably feed on the mixed community found in saguaro rot in the wild. Unlike many other *Caenorhabditis* species, where it is the third larval “dauer” stage (L3d) that actively associate with the phoretic host, host association in *C. drosophilae* is established by second stage larvae (L2) that are attracted to *D. nigrospiracula* pupae ( Kiontke & Sudhaus, 2006). The L2 are predetermined to become L3d and moult just before the adult fly encloses. The L3ds migrate to a pouch in the head of the fly formed by the retracted ptilinum and leave the fly when it visits a cactus rot ( Kiontke, 1997; Kiontke & Sudhaus, 2006). To exit dauer and resume development, *C. drosophilae* requires an unknown signal from the fly ( Kiontke & Sudhaus, 2006).

*C. drosophilae* is most closely related to the formally undescribed *Caenorhabditis* sp. 2, and together these comprise the Drosophilae group ( Dayi *et al.*, 2021; Kiontke *et al.*, 2004; Kiontke *et al.*, 2011). The Drosophilae group was previously included in a larger Drosophilae supergroup ( Dayi *et al.*, 2021; Kiontke *et al.*, 2004; Kiontke *et al.*, 2011), but phylogenetic analysis of whole-genome data suggest that the Drosophilae supergroup is paraphyletic ( Dayi *et al.*, 2021; Sloat *et al.*, 2022; Stevens *et al.*, 2019). To promote the use of nematodes in evolution, ecology, and wider biological research we are sequencing to high quality the genomes of a wide range of species, both free-living and parasitic. Here, we present a chromosome-level reference genome for *C. drosophilae* strain DF5112, an inbred derivative of DF5077, which was isolated from a rotting saguaro cactus in Arizona, USA by Karin Kiontke.

## Genome sequence report

The genome was sequenced from cultured *C. drosophilae* DF5112. We obtained the strain DF5112 from the Caenorhabditis Genetics Center (CGC). A total of 58-fold coverage in Pacific Biosciences single-molecule HiFi long reads was generated. Primary assembly contigs were scaffolded with chromosome conformation Hi-C data. Manual assembly curation corrected 15 missing joins or mis-joins, reducing the scaffold number by 37.5%, and increasing the scaffold N50 by 2.9%.

**Figure 1.**
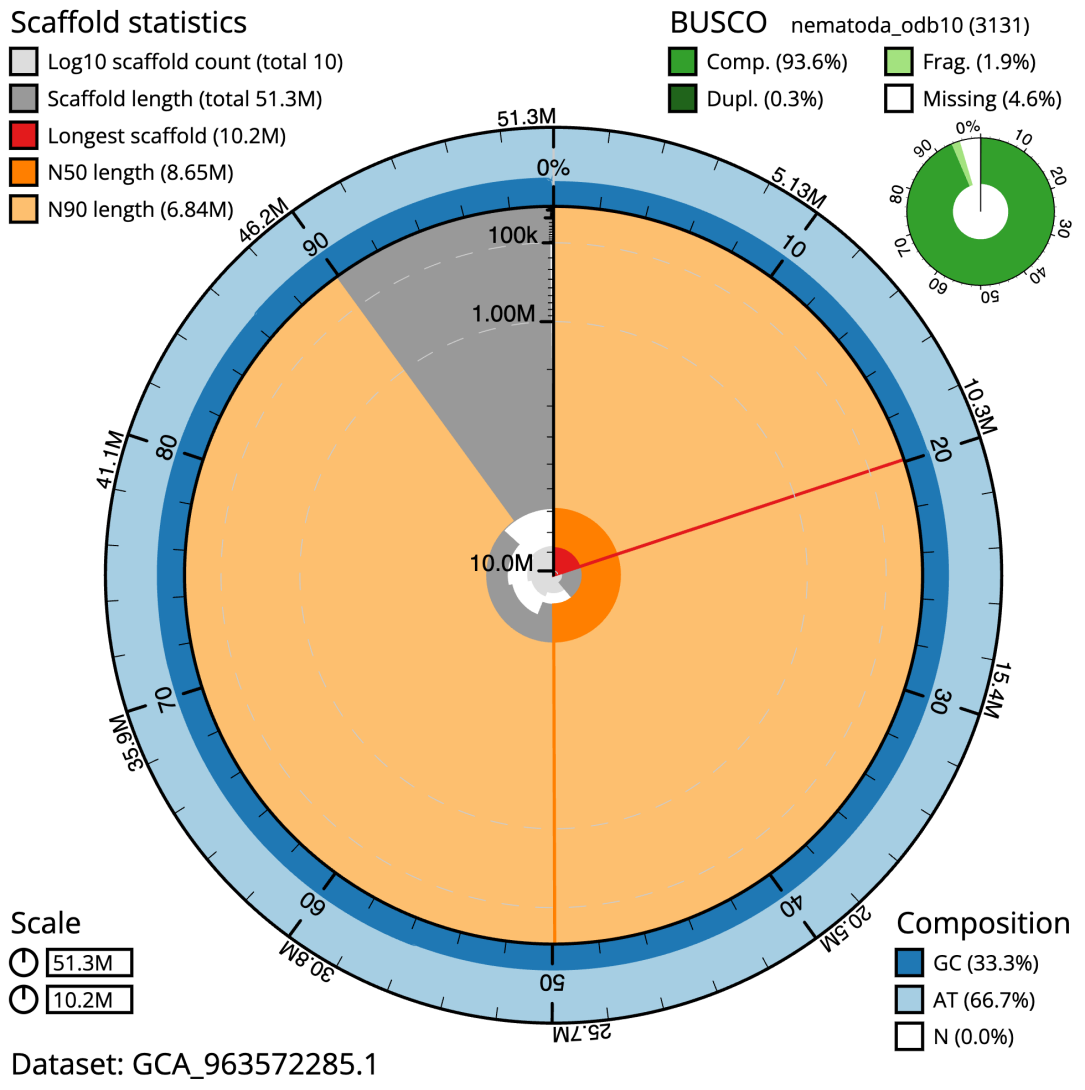
Genome assembly of *Caenorhabditis drosophilae*, nxCaeDros1.1: metrics. The BlobToolKit snail plot shows N50 metrics and BUSCO gene completeness. The main plot is divided into 1,000 size-ordered bins around the circumference with each bin representing 0.1% of the 51,328,830 bp assembly. The distribution of scaffold lengths is shown in dark grey with the plot radius scaled to the longest scaffold present in the assembly (10,235,040 bp, shown in red). Orange and pale-orange arcs show the N50 and N90 scaffold lengths (8,652,941 and 6,839,909 bp), respectively. The pale grey spiral shows the cumulative scaffold count on a log scale with white scale lines showing successive orders of magnitude. The blue and pale-blue area around the outside of the plot shows the distribution of GC, AT and N percentages in the same bins as the inner plot. A summary of complete, fragmented, duplicated and missing BUSCO genes in the nematoda_odb10 set is shown in the top right. An interactive version of this figure is available at https://blobtoolkit.genomehubs.org/view/Caenorhabditis%20drosophilae/dataset/GCA_963572285.1/snail.

**Figure 2.**
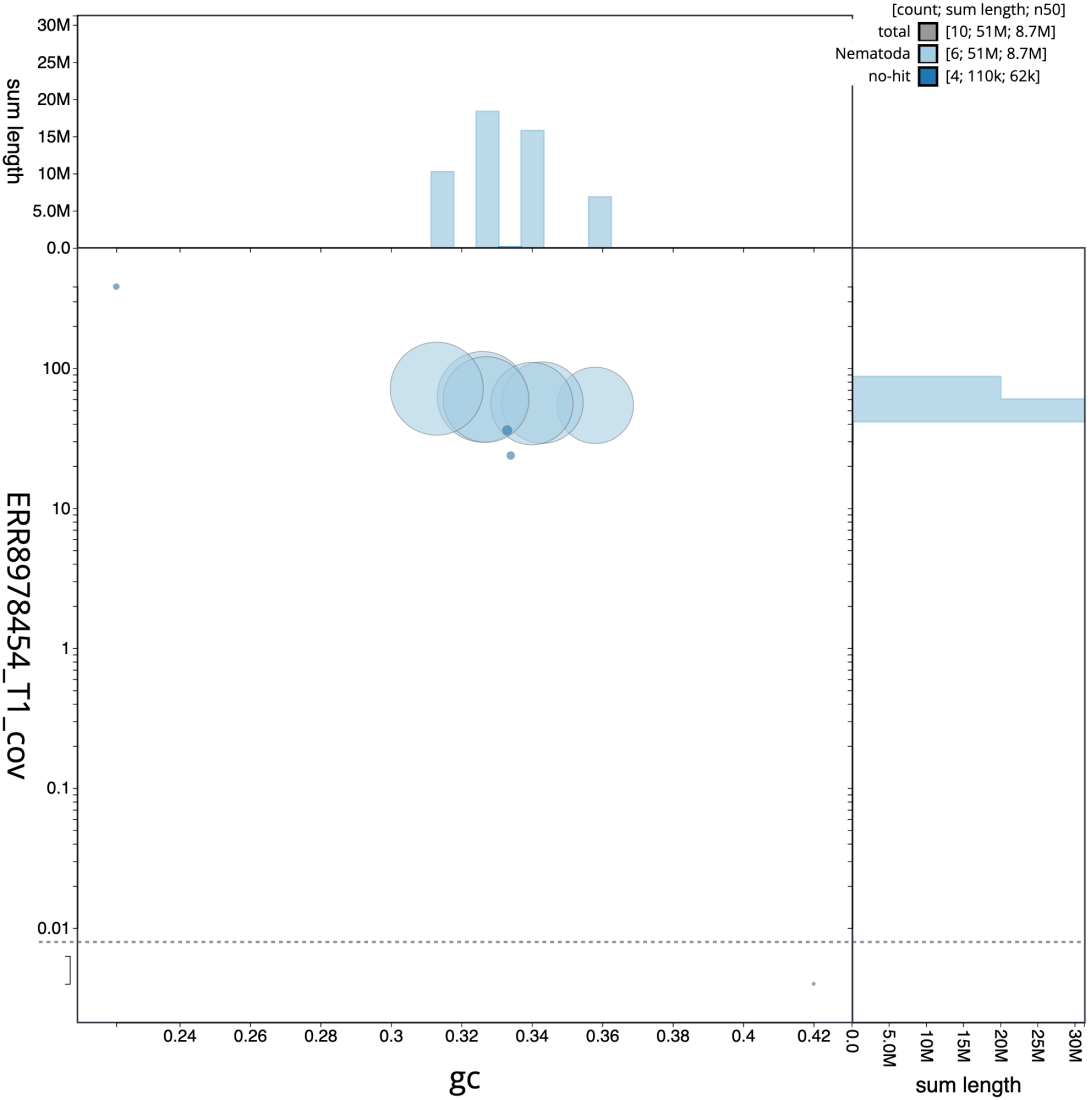
Genome assembly of *Caenorhabditis drosophilae*, nxCaeDros1.1: BlobToolKit GC-coverage plot. Sequences are coloured by phylum. Circles are sized in proportion to sequence length. Histograms show the distribution of sequence length sum along each axis. An interactive version of this figure is available at https://blobtoolkit.genomehubs.org/view/Caenorhabditis%20drosophilae/dataset/GCA_963572285.1/blob.

**Figure 3.**
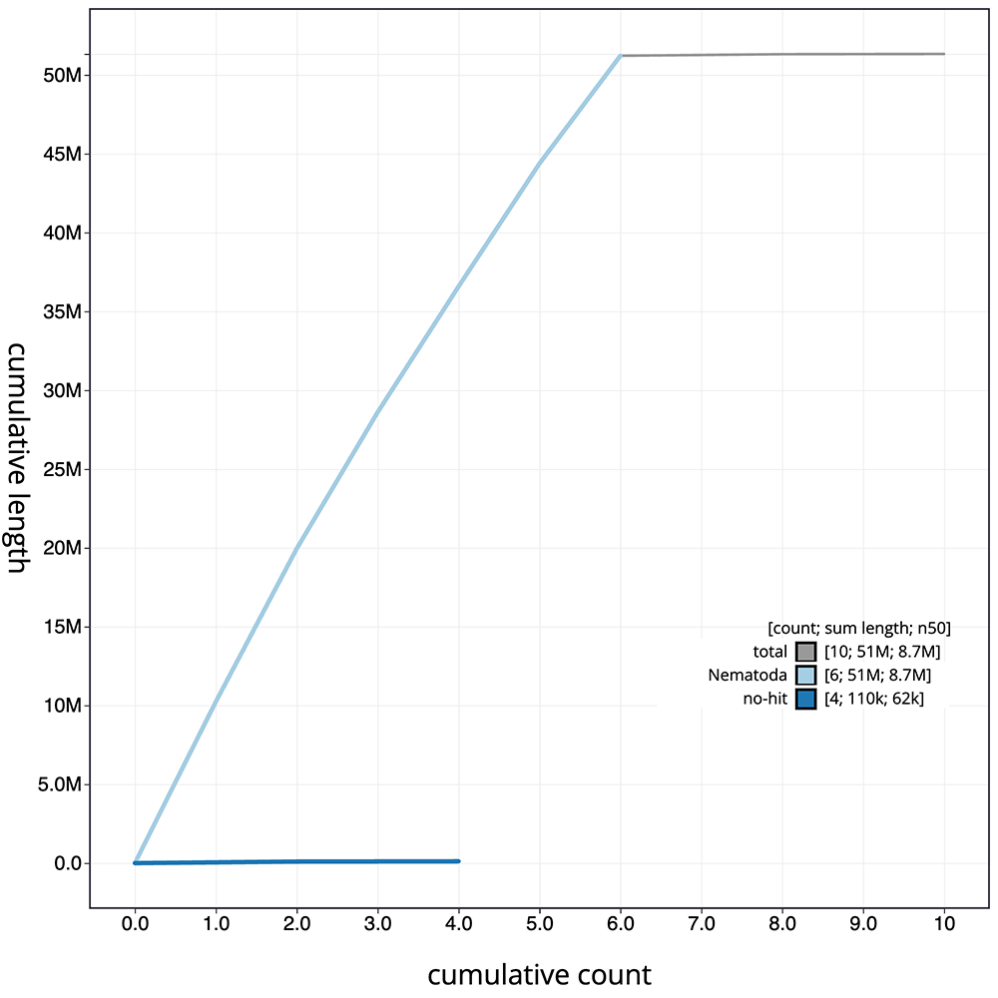
Genome assembly of *Caenorhabditis drosophilae* nxCaeDros1.1: BlobToolKit cumulative sequence plot. The grey line shows cumulative length for all sequences. Coloured lines show cumulative lengths of sequences assigned to each phylum using the buscogenes taxrule. An interactive version of this figure is available at https://blobtoolkit.genomehubs.org/view/Caenorhabditis%20drosophilae/dataset/GCA_963572285.1/cumulative.

**Figure 4.**
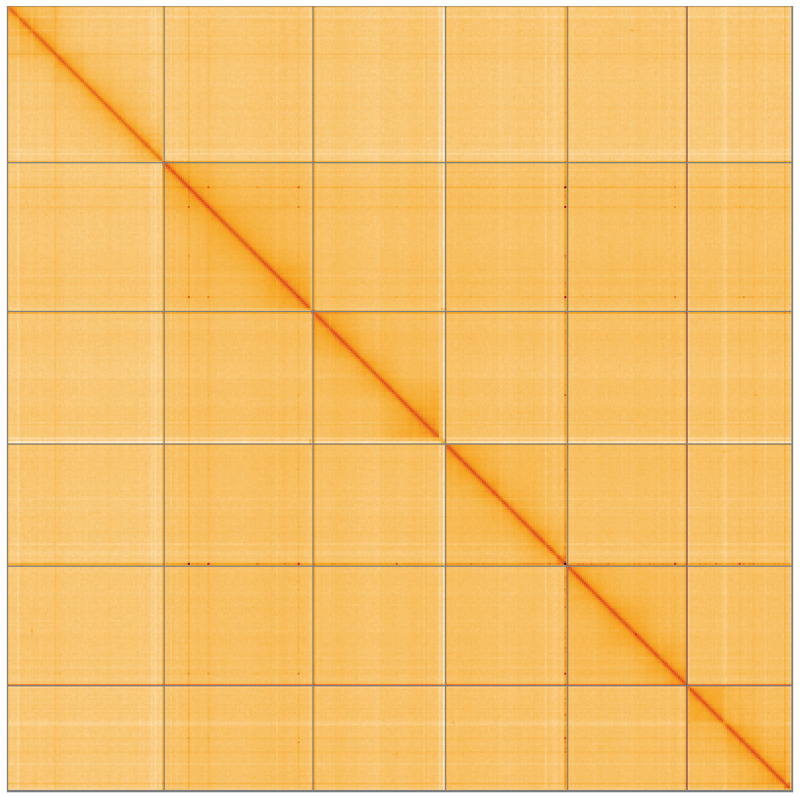
Genome assembly of *Caenorhabditis drosophilae* nxCaeDros1.1: Hi-C contact map of the nxCaeDros1.1 assembly, visualised using HiGlass. Chromosomes are shown in order of size from left to right and top to bottom. An interactive version of this figure may be viewed at https://genome-note-higlass.tol.sanger.ac.uk/l/?d=YVko6Y-FRiWq1BLVluzh8A.

The final assembly has a total length of 51.33 Mb in 10 sequence scaffolds with a scaffold N50 of 8.7 Mb ( Table 1). The snail plot in Figure 1 provides a summary of the assembly statistics, while the distribution of assembly scaffolds on GC proportion and coverage is shown in Figure 2. The cumulative assembly plot in Figure 3 shows curves for subsets of scaffolds assigned to different phyla. Most (99.77%) of the assembly sequence was assigned to 6 chromosomal-level scaffolds, representing 5 autosomes and the X sex chromosome. Chromosome-scale scaffolds were confirmed by the Hi-C data ( Figure 4; Table 2). Chromosomes I_II and II_I have been named to represent a reciprocal translocation between chromosomes I and II in C. *drosophilae* when compared to *C. elegans,* whose chromosome nomenclature has been used to name this assembly.

**Table 1.** Genome data for *Caenorhabditis drosophilae*, nxCaeDros1.1.

Project accession data		
Assembly identifier	nxCaeDros1.1	
Species	*Caenorhabditis drosophilae*	
Specimen	nxCaeDros1	
NCBI taxonomy ID	96641	
BioProject	PRJEB67483	
BioSample ID	SAMEA7996541	
Isolate information	nxCaeDros1, cultured and washed, 150 mg pellet	
Assembly metrics *		*Benchmark*
Consensus quality (QV)	55.6	*≥ 50*
*k*-mer completeness	99.99%	*≥ 95%*
BUSCO **	C:93.3%[S:93.0%,D:0.3%], F:2.2%,M:4.5%,n:3,131	*C ≥ 95%*
Percentage of assembly mapped to chromosomes	99.77%	*≥ 95%*
Sex chromosomes	XX	*localised homologous pairs*
Organelles	Mitochondrial genome: 15.18 kb	*complete single alleles*
Raw data accessions		
PacificBiosciences Sequel II	ERR8978454	
Hi-C Illumina	ERR5762198	
Genome assembly		
Assembly accession	GCA_963572285.1	
*Accession of alternate haplotype*	GCA_963572295.1	
Span (Mb)	51.3	
Number of contigs	46	
Contig N50 length (Mb)	3.5	
Number of scaffolds	9	
Scaffold N50 length (Mb)	8.7	
Longest scaffold (Mb)	10.24	

* Assembly metric benchmarks are adapted from column VGP-2020 of “Table 1: Proposed standards and metrics for defining genome assembly quality” from Rhie *et al.* (2021). ** BUSCO scores based on the nematoda_odb10 BUSCO set using version v5.4.3. C = complete [S = single copy, D = duplicated], F = fragmented, M = missing, n = number of orthologues in comparison. A full set of BUSCO scores is available at https://blobtoolkit.genomehubs.org/view/Caenorhabditis%20drosophilae/dataset/GCA_963572285.1/busco.

**Table 2.** Chromosomal pseudomolecules in the genome assembly of *Caenorhabditis drosophilae*, nxCaeDros1.

INSDC accession	Name	Length (MB)	GC Percent
OY751561.1	II_I	9.71	32.5
OY751560.1	I_II	6.84	36
OY751562.1	III	7.8	34.5
OY751563.1	IV	7.98	34
OY751564.1	V	8.65	32.5
OY751565.1	X	10.24	31.5
OY751566.1	MT	0.02	22

**Figure 5.**
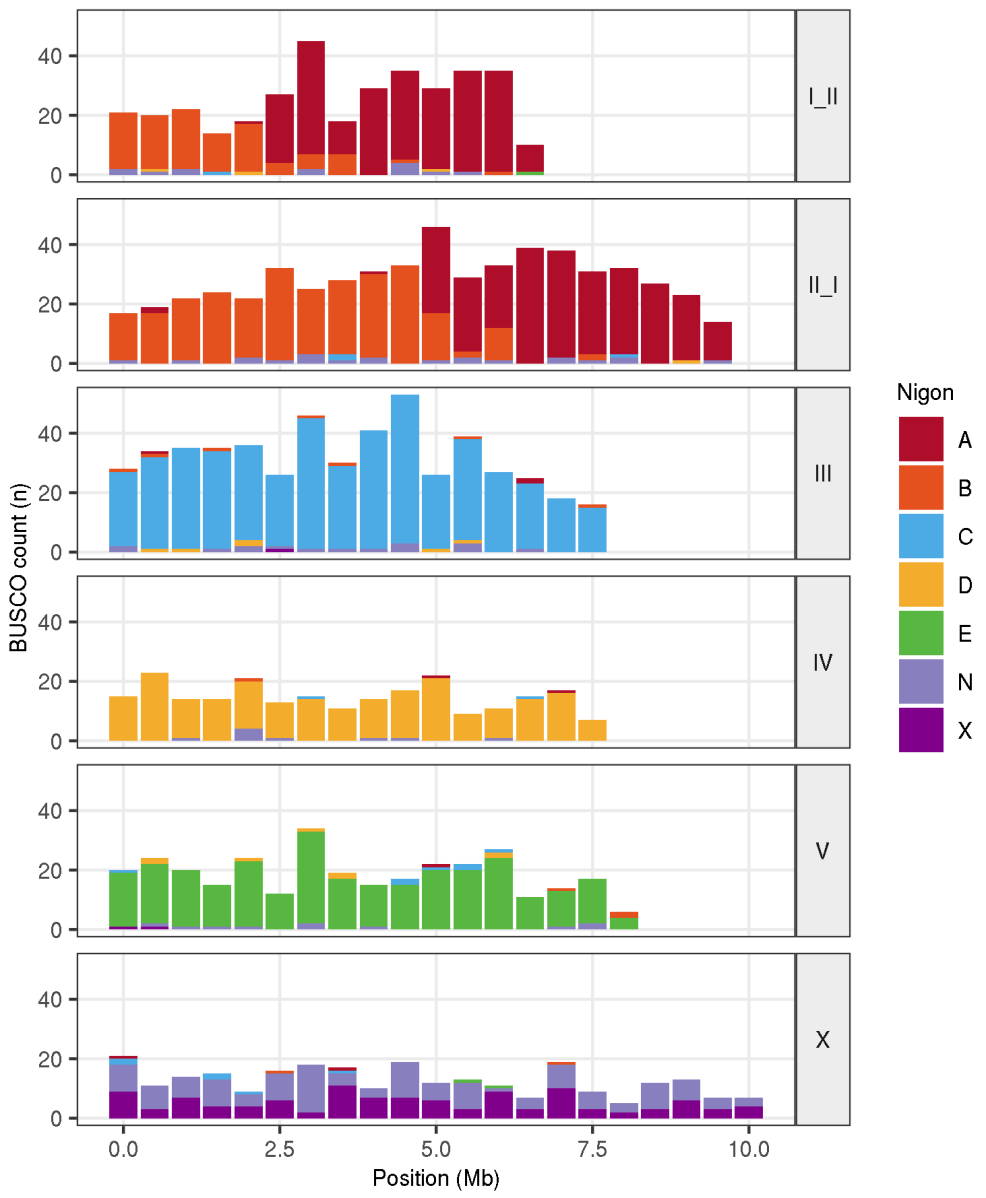
Nigon painting of *Caenorhabditis drosophilae*. Counts of Benchmarking using Single Copy Orthologues (BUSCO) loci in 500 kb windows in the six *C. drosophilae* chromosomes coloured by their allocation to the seven Nigon elements (A-E, N, X) ( Gonzalez de la Rosa *et al.*, 2021).

We mapped the six *C. drosophilae* chromosomes to the rhabditid nematode ancestral linkage groups (Nigon elements) ( Gonzalez de la Rosa *et al.*, 2021) ( Figure 5). The X chromosome is the product of a fusion between NigonN and NigonX that occurred in the last common ancestor of all *Caenorhabditis* species ( Gonzalez de la Rosa *et al.*, 2021). NigonA and NigonB (corresponding to *C. elegans* chromosomes I and II, respectively) have undergone a reciprocal translocation event. The remaining chromosomes correspond to complete Nigon elements, suggesting they have not been involved in fusion or fission events since the last common rhabditid ancestor.

While not fully phased, the assembly deposited is of one haplotype. Contigs corresponding to additional haplotypes present in the strain have also been deposited. The mitochondrial genome was also assembled and can be found as a contig within the multifasta file of the genome submission.

The estimated Quality Value (QV) of the final assembly is 55.6 with *k*-mer completeness of 99.99%, and the assembly has a BUSCO v5.5.0 completeness of 93.3% (single = 93.0%, duplicated = 0.3%), using the nematoda_odb10 reference set ( *n* = 3,131).

## Methods

### Nematode culturing

We obtained *C. drosophilae* DF5112 from the *Caenorhabditis* Genetics Center (CGC). DF5112 is an inbred derivative of DF5077, which was isolated from a rotting saguaro cactus in Arizona, USA by Karin Kiontke. We cultured DF5112 on NGM plates seeded with *Escherichia coli* strain HB101. After most of the bacteria had been consumed, we harvested the nematodes by washing the plates with cold M9 into 50 ml Falcon tubes, which we then centrifuged at 4000 rcf for 8 min with the brake set to the value of 3. We discarded the supernatant and washed the nematodes a further three times using M9 supplemented with 0.01% Tween. We performed a final wash using PBS buffer. We divided the worms into 1.5 ml DNA LoBind ^®^ Tubes (Eppendorf). We recorded the weight of each pellet before flash freezing in liquid nitrogen and storing at –70°C.

### Long-read DNA extraction and sequencing

We extracted high molecular weight DNA from a 150 mg pellet of nematodes using the MagAttract HMW DNA kit (Qiagen) with the following modifications. The lysis mix was prepared and placed on ice: 200 µl PBS, 20 µl ProteinaseK (Qiagen), 4 µl RNase A (Qiagen), 150 µl AL buffer (Qiagen). We added 75 µl of the lysis buffer mix to the frozen nematode pellet and used a BioMasher II to disrupt the pellet. We added the remaining lysis buffer and mixed with a wide bore tip. We transferred the lysis solution to a 2 ml DNA LoBind ^®^ Tube (Eppendorf) and digested overnight at 45°C mixing at 600 rpm in a ThermoMixer C (Eppendorf). We added 15 µl of MagAttract Suspension G before we mixed everything with 280 µl Buffer MB. We eluted the MagAttract beads twice using 200 µl of Buffer AE in each elution step. We incubated the second elution mix at 25°C with 1000 rpm for 3 min in the ThermoMixer C before transferring the elution liquid to a new 1.5 ml LoBind microtube. In total, we extracted 2,700 ng high molecular weight DNA. 1,530 ng of this DNA was sheared to an average size of 13.2 kb with a Megaruptor 3 (setting 30) (Diagenode). The sheared DNA was SPRI cleaned with 1.8x of AMPure XP beads (Beckman Coulter).

A PacBio library was prepared from the extracted DNA by the Scientific Operations: Sequencing Operations core at the Wellcome Sanger Institute using the PacBio Low DNA Input Library Preparation Using SMRTbell Express Template Prep Kit 2.0. The library was sequenced on a single PacBio Sequel IIe flow cell.

### Hi-C sequencing

Hi-C library preparation and sequencing were performed by the Scientific Operations: Sequencing Operations core at the Wellcome Sanger Institute. A 20 mg pellet of mixed stage nematodes was processed using the Arima Hi-C version 2 kit following the manufacturer’s instructions. An Illumina library was prepared using the NEBNext Ultra II DNA Library Prep Kit and sequenced on one-eighth of a NovaSeq S4 lane using paired-end 150 bp sequencing.

### Genome assembly, curation and evaluation

We removed adapter sequences from the PacBio HiFi data using HiFiAdapterFilt ( Sim *et al.,* 2022). We used Jellyfish 2.3.0 ( Marçais & Kingsford, 2011) to count *k*-mers of length 31 in each read set and GenomeScope 2.0 ( Ranallo-Benavidez *et al.*, 2020) to estimate genome size and heterozygosity. We first generated a primary and alternate assembly from the PacBio HiFi data using Hifiasm ( Cheng *et al.*, 2021). We randomly subsampled 10% of the Hi-C reads using samtools 1.14 ( Danecek *et al.*, 2021) and aligned them to the hifiasm primary assembly using bwa-mem 0.7.17-r1188 ( Li, 2013), filtered out PCR duplicates using picard 2.27.1–0 (available at http://broadinstitute.github.io/picard/), and scaffolded the assembly using YaHS ( Zhou *et al.*, 2023). We ran BlobToolKit 2.6.5 ( Challis *et al.*, 2020) on the scaffolded assembly and used the interactive web viewer to manually screen for scaffolds derived from non-target organisms. We also ran BlobToolKit on an unscaffolded version of the alternate assembly. We removed one and three Proteobacteria ( *E. coli*) contigs from the primary and alternate assemblies, respectively. After removing contaminants, we used MitoHiFi 2.2 ( Uliano-Silva *et al.*, 2023) to extract and annotate the mitochondrial genome. We removed residual haplotypic duplication from each assembly using purge_dups ( Guan *et al.*, 2020) and scaffolded the purged primary assembly using Hi-C data ( Rao *et al.*, 2014), as previously described.

The assembly was checked for contamination and corrected using the gEVAL system ( Chow *et al.*, 2016) as described previously ( Howe *et al.*, 2021). Manual curation was performed using gEVAL, HiGlass ( Kerpedjiev *et al.*, 2018) and PretextView ( Harry, 2022). The mitochondrial genome was assembled using MitoHiFi ( Uliano-Silva *et al.*, 2023), which runs MitoFinder ( Allio *et al.*, 2020) or MITOS ( Bernt *et al.*, 2013) and uses these annotations to select the final mitochondrial contig and to ensure the general quality of the sequence.

### Evaluation of final assembly

The final assembly was post-processed and evaluated with the three Nextflow ( Di Tommaso *et al.*, 2017) DSL2 pipelines “sanger-tol/readmapping” ( Surana *et al.*, 2023a), “sanger-tol/genomenote” ( Surana *et al.*, 2023b), and “sanger-tol/blobtoolkit” ( Muffato *et al.*, 2024). The pipeline sanger-tol/readmapping aligns the Hi-C reads with bwa-mem2 ( Vasimuddin *et al.*, 2019) and combines the alignment files with SAMtools ( Danecek *et al.*, 2021). The sanger-tol/genomenote pipeline transforms the Hi-C alignments into a contact map with BEDTools ( Quinlan & Hall, 2010) and the Cooler tool suite ( Abdennur & Mirny, 2020), which is then visualised with HiGlass ( Kerpedjiev *et al.*, 2018). It also provides statistics about the assembly with the NCBI datasets ( Sayers *et al.*, 2024) report, computes *k*-mer completeness and QV consensus quality values with FastK and MerquryFK, and a completeness assessment with BUSCO ( Manni *et al.*, 2021).

The sanger-tol/blobtoolkit pipeline is a Nextflow port of the previous Snakemake Blobtoolkit pipeline ( Challis *et al.*, 2020). It aligns the PacBio reads with SAMtools and minimap2 ( Li, 2018) and generates coverage tracks for regions of fixed size. In parallel, it queries the GoaT database ( Challis *et al.*, 2023) to identify all matching BUSCO lineages to run BUSCO ( Manni *et al.*, 2021). For the three domain-level BUSCO lineage, the pipeline aligns the BUSCO genes to the Uniprot Reference Proteomes database ( Bateman *et al.*, 2023) with DIAMOND ( Buchfink *et al.*, 2021) blastp. The genome is also split into chunks according to the density of the BUSCO genes from the closest taxonomically lineage, and each chunk is aligned to the Uniprot Reference Proteomes database with DIAMOND blastx. Genome sequences that have no hit are then chunked with seqtk and aligned to the NT database with blastn ( Altschul *et al.*, 1990). All those outputs are combined with the blobtools suite into a blobdir for visualisation.

All three pipelines were developed using the nf-core tooling ( Ewels *et al.*, 2020), use MultiQC ( Ewels *et al.*, 2016), and make extensive use of the Conda package manager, the Bioconda initiative ( Grüning *et al.*, 2018), the Biocontainers infrastructure ( da Veiga Leprevost *et al.*, 2017), and the Docker ( Merkel, 2014) and Singularity ( Kurtzer *et al.*, 2017) containerisation solutions.

Table 3 contains a list of relevant software tool versions and sources.

**Table 3.** Software tools: versions and sources.

Software tool	Version	Source
BEDTools	2.30.0	https://github.com/arq5x/bedtools2
Blast	2.14.0	ftp://ftp.ncbi.nlm.nih.gov/blast/executables/blast+/
BlobToolKit	4.3.7	https://github.com/blobtoolkit/blobtoolkit
BUSCO	5.4.3	https://gitlab.com/ezlab/busco
BUSCO	5.4.3 and 5.5.0	https://gitlab.com/ezlab/busco
bwa-mem2	2.2.1	https://github.com/bwa-mem2/bwa-mem2
Cooler	0.8.11	https://github.com/open2c/cooler
DIAMOND	2.1.8	https://github.com/bbuchfink/diamond
fasta_windows	0.2.4	https://github.com/tolkit/fasta_windows
FastK	427104ea91c78c3b8b8b49f1a7d6bbeaa869ba1c	https://github.com/thegenemyers/FASTK
GoaT CLI	0.2.5	https://github.com/genomehubs/goat-cli
Hifiasm	0.16.1-r375	https://github.com/chhylp123/hifiasm
HiGlass	1.11.6	https://github.com/higlass/higlass
HiGlass	44086069ee7d4d3f6f3f0012569789ec138f42b84aa44357826c0b6753eb28de	https://github.com/higlass/higlass
Jellyfish	2.3.0	https://github.com/gmarcais/Jellyfish/releases
MerquryFK	d00d98157618f4e8d1a9190026b19b471055b22e	https://github.com/thegenemyers/MERQURY.FK
MitoHiFi	2	https://github.com/marcelauliano/MitoHiFi
MultiQC	1.14, 1.17, and 1.18	https://github.com/MultiQC/MultiQC
NCBI Datasets	15.12.0	https://github.com/ncbi/datasets
Nextflow	23.04.0-5857	https://github.com/nextflow-io/nextflow
PretextView	0.2	https://github.com/wtsi-hpag/PretextView
purge_dups	1.2.5	https://github.com/dfguan/purge_dups
samtools	1.16.1, 1.17, and 1.18	https://github.com/samtools/samtools
sanger-tol/genomenote	1.1.1	https://github.com/sanger-tol/genomenote
sanger-tol/readmapping	1.2.1	https://github.com/sanger-tol/readmapping
Seqtk	1.3	https://github.com/lh3/seqtk
Singularity	3.9.0	https://github.com/sylabs/singularity
TreeVal	1.0.0	https://github.com/sanger-tol/treeval
YaHS	1.1a	https://github.com/c-zhou/yahs

## Data Availability

European Nucleotide Archive: *Caenorhabditis drosophilae* genome assembly, nxCaeDros1. Accession number PRJEB67483; https://www.ebi.ac.uk/ena/browser/view/PRJEB67483. The genome sequence is released openly for reuse. The *Caenorhabditis drosophilae* genome sequencing initiative is part of the 959 Nematode Genomes project. All raw sequence data and the assembly have been deposited in INSDC databases. Raw data and assembly accession identifiers are reported in Table 1.

## Author information

Members of the Wellcome Sanger Institute Tree of Life Management, Samples and Laboratory team are listed here: https://doi.org/10.5281/zenodo.10066175.

Members of Wellcome Sanger Institute Scientific Operations: Sequencing Operations are listed here: https://doi.org/10.5281/zenodo.10043364.

Members of the Wellcome Sanger Institute Tree of Life Core Informatics team are listed here: https://doi.org/10.5281/zenodo.10066637.
