# Supplementary material for: The genome sequence of the nematode Caenorhabditis drosophilae (Rhabditida, Rhabditidae) (Kiontke, 1997)
Source: Wellcome Open Res. 2024 May 24;9:292. [Version 1] doi: 10.12688/wellcomeopenres.22416.1 (PMC11303941; doi:10.12688/wellcomeopenres.22416.1)
Supplement: Peer review history

# Reviewer report: Reviewer response for version 1

**DOI:** 10.21956/wellcomeopenres.24695.r89264

**Published:** 2024-08-09

**Authors:** Gavin C. Woodruff

**Affiliations:** 1 School of Biological Sciences, The University of Oklahoma, Norman, Oklahoma, USA

Here, the existence of a *Caenorhabditis drosophilae* genome assembly is communicated and shared. The assembly appears to be of high quality and completeness. I am very excited to see this assembly shared with the community, and I am happy there is now a citable source associated with this assembly. Below are my thoughts emerging from the request for peer review.

 1. It appears that this is the report of an assembly alone. Annotations are conspicuously absent. However, it appears the annotations might exist, as the Figure 5 legend notes the use of single-copy orthologs. But, from the methods, it is not obvious to me how single-copy orthologs were inferred. Additionally, the BUSCO-associated methods refer to "blastp," which requires amino acid sequences (and suggests the existence of annotations). However, it is possible that the approaches used to generate Figure 5 (and the BUSCO completeness scores) are entirely annotation-independent. Beyond this, as the assembly size is super small for a *Caenorhabditis* species (51.3 MB), it is natural to wonder how many genes are present. Could this be the smallest *Caenorhabditis* genome yet? I suspect it may be, as it is smaller than the 59 MB *C. niphades* assembly! Yet, in this emerging era of preprints, micropublications, and data notes, I feel somewhat uncomfortable making a comment like this. Nonetheless, the lack of annotations makes this resource less useful and more difficult to contextualize than it would be otherwise.

 Note, if the BUSCO scores and Nigon painting results were generated in an annotation-independent manner (or if I just overlooked the existence of available annotations), this comment can be ignored.

 2. The reported methods and assembly statistics are transparent and sufficient for the aims of a data note. Table 3 is fantastic. But, I do not believe I see any links to the code used to generate this assembly? While I do not believe this is strictly required for a data note like this, I believe this would be helpful for those who want to generate nematode assemblies in a similar manner. Again, if I overlooked this link in the publication, this comment can be ignored.

 3. I assume the bottom y-axis of Figure 2 corresponds to coverage because "_cov" is on the label. If this is noting something else, then this axis label should be changed.

 4. This is very minor point. But, while blobtoolkit seems like a fantastic tool for evaluating genome assemblies, Figure 1 is not exactly intuitive to me. Multiple two-axis Cartesian plots are preferable to a single, information-dense circle. However, I understand the aim here is to just *get this out*, so this figure is sufficient for this paper.

 And that's it— thanks for assembling this genome and sharing it with the community!

Are sufficient details of methods and materials provided to allow replication by others?

Yes

Is the rationale for creating the dataset(s) clearly described?

Yes

Are the datasets clearly presented in a useable and accessible format?

Yes

Are the protocols appropriate and is the work technically sound?

Yes

Reviewer Expertise:

Genetics, evolution, development, and genomics. I have never personally assembled a genome for publication, although I have published work in comparative genomics.

I confirm that I have read this submission and believe that I have an appropriate level of expertise to confirm that it is of an acceptable scientific standard.

# Reviewer report: Reviewer response for version 1

**DOI:** 10.21956/wellcomeopenres.24695.r89257

**Published:** 2024-08-06

**Authors:** Xue Qing

**Affiliations:** 1 Nanjing Agricultural University, Nanjing, China

This MS describes the genome sequencing and assembly for Caenorhabditis drosophilae. The manuscript is well written, and genome was properly assembled and analyzed. The generation of chromosomal level genome is a major achievement, which is very valuable for the community. It’s a pity author did not sequence the transcriptome or predict the genes. It would be interesting to see the genome composition. However, this may not be necessary for such type of data report.

**Comments:**

- In background, author detailed the biology of sequenced species, but lacks the information for genomes in Caenorhabditis (how many been sequenced to chromosome level, size range...), or at least for closely related species within Drosophilae group. In fact, one of very important result for me is the small genome size, which Is half of C.elegans.
- The Table 1, figure1 and 3 are redundant, presenting the nearly same information. It would be interesting to make a table to show the genome statistics in genus Caenorhabditis, or the a phylogenomic tree showing the placement of Caenorhabditis drosophilae. Alternatively, you may replace one of this figure to GenomeScope graph, or synteny analysis in compare to C.elegans/other species in Drosophilae group

**Other minor issues:**

- In MS, it was stated the final assembly has a total length of 51.33 Mb in 10 sequence scaffolds, but in table 1 it was 9 scaffolds
- There are many mistakes in table3,  e.g. two HiGlass and BUSCO in table 3, the Blast should be capitalized. In text the BlobToolKit is in version 2.6.5 but table 4.3.7. I haven't check them, but please carefully check to make sure they are correct.

Are sufficient details of methods and materials provided to allow replication by others?

Yes

Is the rationale for creating the dataset(s) clearly described?

Yes

Are the datasets clearly presented in a useable and accessible format?

Yes

Are the protocols appropriate and is the work technically sound?

Yes

Reviewer Expertise:

genomics, diversity, evolution, Nematodes

I confirm that I have read this submission and believe that I have an appropriate level of expertise to confirm that it is of an acceptable scientific standard.

# Reviewer report: Reviewer response for version 1

**DOI:** 10.21956/wellcomeopenres.24695.r89265

**Published:** 2024-08-06

**Authors:** Asher D. Cutter

**Affiliations:** 1 University of Toronto, Toronto, Ontario, Canada

Kieninger et al. present in a Data Note the genome sequencing and assembly for the nematode species Caenorhabditis drosophilae. This new chromosome-scale genome assembly will be a valuable contribution to the broader research community. Overall, the data collection and analysis appears sound with standard procedures and tools that are available to the research community, the links to which are provided in a table. The presentation also is clear. I have no major concerns or major criticisms. 

**Minor correction**:

 I noticed what appears to be a typo for a species name of the host insect in the first sentence of the Background that should be corrected (nigrospiculata -> nigrospiracula).

Are sufficient details of methods and materials provided to allow replication by others?

Yes

Is the rationale for creating the dataset(s) clearly described?

Yes

Are the datasets clearly presented in a useable and accessible format?

Yes

Are the protocols appropriate and is the work technically sound?

Yes

Reviewer Expertise:

Evolutionary genomics

I confirm that I have read this submission and believe that I have an appropriate level of expertise to confirm that it is of an acceptable scientific standard.

# Reviewer report: Reviewer response for version 1

**DOI:** 10.21956/wellcomeopenres.24695.r87929

**Published:** 2024-08-06

**Authors:** Komal Kumar Bollepogu Raja

**Affiliations:** 1 Department of Pathology & Immunology, Baylor College of Medicine, Houston, TX, USA

The article by Kieninger et al, provides a comprehensive genomic analysis of a nematode species *C. drosophilae*. This study highlights the organism's genetic structure and assembly methodology. The authors show that the final assembly spans 51.33 Mb across 10 scaffolds, with a notable scaffold N50 of 8.7 Mb. The genomic completeness is robust, with a Quality Value (QV) of 55.6, k-mer completeness of 99.99%, and BUSCO assessment showing 93.3% completeness against the nematoda_odb10 reference set. The authors have leveraged bioinformatics tools such as BlobToolKit for genome evaluation, MitoHiFi for mitochondrial genome annotation, and gEVAL for contamination screening and correction. Their analyses have revealed evolutionary insights, including a fusion event on the X chromosome and reciprocal translocations between chromosomes I and II, compared to the model nematode *C. elegans*.

 Overall, this article is well written and advances understanding of *C. drosophilae*'s genomic architecture but also sets methodological standards for future comparative genomic studies within the *Caenorhabditis* genus. Most of my comments are minor and only aim at improving the manuscript.

**Minor comments**

- The article could benefit from providing more context about why *C. drosophilae* was chosen for genome sequencing and its significance in evolutionary or ecological studies. A brief introduction situating the species within the broader context of nematode biology would enhance the relevance of the study. The Background section could be expanded to provide more context.
- A section discussing the implications of the genome assembly could provide insights into how this research contributes to understanding nematode evolution, ecology, or other biological aspects
- Please add more detail to some figure legends for a broader audience in biology.

Are sufficient details of methods and materials provided to allow replication by others?

Yes

Is the rationale for creating the dataset(s) clearly described?

Yes

Are the datasets clearly presented in a useable and accessible format?

Yes

Are the protocols appropriate and is the work technically sound?

Yes

Reviewer Expertise:

Single cell omics, Genomics, NGS, Bioinformatics

I confirm that I have read this submission and believe that I have an appropriate level of expertise to confirm that it is of an acceptable scientific standard.

# Reviewer report: Reviewer response for version 1

**DOI:** 10.21956/wellcomeopenres.24695.r89261

**Published:** 2024-08-05

**Authors:** Christian Rödelsperger

**Affiliations:** 1 Department for Integrative Evolutionary Biology, Max Planck Institute for Biology, Tübingen, Germany

The manuscript by Kieninger et al. present the genome assembly of nematode Caenorhabditis drosophilae from PacBio HiFi read and Hi-C data. Based on standard metrics, like N50 or BUSCO, the assembly seems to be of really high quality.

 I have indicated that the description of the methods only partially allow replication, because it appears that specific command line parameters are not mentioned for various softwares and because the manuscript describes manual curations. The latter is not really an issue because I am sure that the manual curations let to some improvements of the final assembly. However, unless all the changes have been documented or access to the raw assembly is provided, this makes the reproducibility of the complete assembly process a bit difficult. However, I do not see that as a real problem and I would leave it to the authors if and how to address this. 

**Specific comments**

- "Chromosome-scale scaffolds were confirmed by the Hi-C data (Figure 4; Table 2)."   ->

Calling this "confirmation" is a bit circular since the scaffolds were generated using the Hi-C data. My suggestion: "Figure 4 shows the chromosomal layout of the C. drosophilae genome as inferred from Hi-C data."

**Figure 4**

-  This Figure lacks a color scale and an indication of how much sequence a single dot represents.

** Methods**

- " We cultured DF5112 on NGM plates seeded with Escherichia coli strain HB101."

An explanation why C. drosophilae was not cultured on OP50 would be nice. Does that go back to the initial work by Karin Kiontke?

**Methods**

- The authors should indicate the command line parameters for various employed programs or indicate the default options were used.

**Methods**

- "We removed residual haplotypic duplication from each assembly using purge_dups (Guan et al., 2020) and scaffolded the purged primary assembly using Hi-C data (Rao et al., 2014), as previously described."  

It is not clear to me to what "as previously described" refers to. Does Rao et al. 2014 refer to the method that was used for scaffolding? Is that different from YaHS?

 The section "Evaluation of final assembly" describes a computational pipeline. As I am not completely familiar with all the software, I would greatly appreciate a small description what useful information could be gained by running these programs. I think, what would be also helpful, would be a statement of how these results looked like for the C. drosophilae genome, e.g. how does the coverage distribution looks like across the genome? Is the X:autosome coverage ratio as expected?

 Figure 5, just out of curiosity, I wonder if it the reciprocal translocations between chromosomes I and II are shared with any other published Caenorhabditis genomes or did this happen quite recently in the lineage leading to C. drosophilae?

Are sufficient details of methods and materials provided to allow replication by others?

Partly

Is the rationale for creating the dataset(s) clearly described?

Yes

Are the datasets clearly presented in a useable and accessible format?

Yes

Are the protocols appropriate and is the work technically sound?

Yes

Reviewer Expertise:

Nematode genomics

I confirm that I have read this submission and believe that I have an appropriate level of expertise to confirm that it is of an acceptable scientific standard.
